# Metallo‐Supramolecular Gels that are Photocleavable with Visible and Near‐Infrared Irradiation

**DOI:** 10.1002/anie.201707321

**Published:** 2017-11-15

**Authors:** Sabrina Theis, Aitziber Iturmendi, Christian Gorsche, Marco Orthofer, Markus Lunzer, Stefan Baudis, Aleksandr Ovsianikov, Robert Liska, Uwe Monkowius, Ian Teasdale

**Affiliations:** ^1^ Institute of Inorganic Chemistry Johannes Kepler University Linz Altenberger Strasse 69 4040 Linz Austria; ^2^ Institute of Polymer Chemistry Johannes Kepler University Linz Altenberger Strasse 69 4040 Linz Austria; ^3^ Institute of Applied Synthetic Chemistry Technische Universität Wien Austria; ^4^ Institute of Materials Science and Technology Technische Universität Wien Austria; ^5^ Austrian Cluster for Tissue Regeneration Austria; ^6^ Linz School of Education Johannes Kepler Universität Linz Austria

**Keywords:** caged compounds, gels, metallopolymers, ruthenium, two-photon absorption

## Abstract

A photolabile ruthenium‐based complex, [Ru(bpy)_2_(4AMP)_2_](PF_6_)_2_, (4AMP=4‐(aminomethyl)pyridine) is incorporated into polyurea organo‐ and hydrogels via the reactive amine moieties on the photocleavable 4AMP ligands. While showing long‐term stability in the dark, cleavage of the pyridine–ruthenium bond upon irradiation with visible or near‐infrared irradiation (in a two‐photon process) leads to rapid de‐gelation of the supramolecular gels, thus enabling spatiotemporal micropatterning by photomasking or pulsed NIR‐laser irradiation

Functional polymers which can be photochemically manipulated by exogenous light have immense promise in materials science, with applications ranging from drug delivery to photolithography.^1^ Photoresponsive polymers^2^ offer localization, either via photo‐isomerization^3^ or photo‐cleavage mechanisms.^4^ Photoresponsive units can be attached as pendant groups or incorporated into the main‐chain of photocleavable polymers,^5^ which degrade to smaller molecules in a light‐driven response. Photocleavable polymers also have significant potential in biological applications in particular spatiotemporal patterning of cellular microenvironment^6^ in tissue engineering,^7^ smart‐drug‐delivery and diagnostic systems.^8^ For such applications, materials are required which are sensitive to longer wavelength, visible and near‐infrared (NIR) irradiation, owing to its deeper penetration and lower risk of damage to biological tissue compared to UV irradiation.^1^, ^9^ Although a number of systems have been proposed which are responsive to long wavelength and NIR, for example, by two‐photon‐absorption processes,^10^ photocleavage is often less efficient for standard organic systems because of a lack of sensitivity to the lower energy provided.

In the search for improved material properties, metallopolymers have become of ever increasing interest^11^ for their potential to supply advanced functional materials for a wide range of applications.^12^ Herein we report a novel ruthenium‐based complex and its incorporation into polyureas to give photocleavable supramolecular metallopolymers. A small number of ruthenium‐containing organic polymers have been reported previously^13^ with interesting properties, for example, in opto‐electronic applications^14^ or as self‐oscillating polymer gels.^15^ Meanwhile *cis*‐[Ru^II^(bpy)_2_(L^1^)(L^2^)]^2+^ complexes have been reported in which a rapid cleavage of the ligands has been shown upon excitation with visible or NIR light.^16^ Such de‐caging systems have been investigated in pro‐drug‐type formulations in which the ligands consist of biologically active molecules.^17^ The elementary photoreaction is fast (in the nanosecond region), has high quantum yields (up to 13 %), can be triggered with visible light at approximately 450 nm, and has been shown to exhibit efficient photocleavage using NIR light.^18^ Furthermore, since similar ruthenium species have been reported to be biologically benign,^16^ such systems may have potential use in biological systems.

We prepared an amino‐functionalized ruthenium complex, [Ru(bpy)_2_(4AMP)_2_]^2+^(PF_6_)_2_ (bpy=2,2′‐bipyridine, 4AMP=4‐(aminomethyl)pyridine), starting from [Ru(bpy)_2_Cl_2_] and 4AMP at 80 °C in H_2_O (Scheme 1). The complex was precipitated as its PF_6_ salt as an orange powder. ^1^H and ^13^C{^1^H} NMR spectroscopy and mass spectrometry were used to confirm the structure of the compound (Figure S1–S3 in the Supporting Information). In the dry, solid state, the complex is stable in the dark, as well as under irradiation with visible light, with no observable changes in the ^1^H NMR spectra upon exposure to sunlight for several months (Figure S4). Furthermore, in acetonitrile solution, the Ru‐complex can be stored for several months in the dark, again without any changes observable by ^1^H NMR spectroscopy (Figure S5). Photochemical characterization showed the functionalized ruthenium complex [Ru(bpy)_2_(4AMP)_2_](PF_6_)_2_ to have a broad long‐wavelength absorption peak with a maximum at 460 nm and a shoulder at around 430 nm (Figure 1 a), which is typical for a metal‐to‐ligand charge transfer (MLCT) and comparable to the spectra of known bipyridine ruthenium(II) complexes.^19^ Further signals can be found at 290 and 338 nm which are due to π–π* transitions within the bipyridine ligands and d–d transitions.^20^ Upon irradiation with light longer than 395 nm in MeCN (conc.≈10^−7^ mol L^−1^) the signal at 460 nm decreases and the MLCT band shifts to shorter wavelength. The UV/Vis absorption spectra suggest the photoreaction is a two‐step process with a successive cleavage of the pyridine ligands at considerably different reaction rates. Initially only one ligand cleaves resulting in the isosbestic point at 442 nm. As the photocleavage progresses this isosbestic point disappears because of overlay from the second cleavage reaction. After approximately 1 min the initial complex is converted almost completely into [Ru(bpy)_2_(4AMP)(MeCN)](PF_6_)_2_, after which a second isosbestic point is established at around 432 nm. The second cleavage is much slower requiring about 15 min to almost completely cleave the 4AMP ligand. Interestingly, the emission intensity decreases with progressing reaction (Figure S6). The photoreaction upon irradiation with visible light was also monitored by ^1^H NMR spectroscopy (Figure S7) and confirm the stepwise photocleavage of the 4AMP ligands, albeit at slower rates owing to the higher concentration of the NMR sample (16.3 mmol L^−1^ compared to the UV/Vis sample 10^−4^ mmol L^−1^). The signals of two of the *ortho*‐protons H_b_ of the bpy ligand could be followed for kinetic analysis (Figure 1 b): For [Ru(bpy)_2_(4AMP)_2_]^2+^ this signal is at 8.95 ppm and shifts to 9.43 ppm upon the first cleavage and to 9.34 ppm upon cleavage of the second ligand. The plot of the integrals of these three signals against time gives the typical profile of a consecutive reaction (Figure 1 c). Cleavage of the initial complex is completed within one hour at this concentration to yield the mono‐substituted complex, whereas cleavage of the second 4AMP ligand is much slower and takes about 20 hours. Photosubstitution of the ligand is also possible in H_2_O. Contrary to acetonitrile a bathochromic shift of the MLCT band can be observed in the UV/Vis absorption spectra upon substitution with H_2_O (Figure S8) and only one 4AMP ligand is cleaved according to ^1^H NMR spectroscopy (Figure S9).

**Figure 1:**
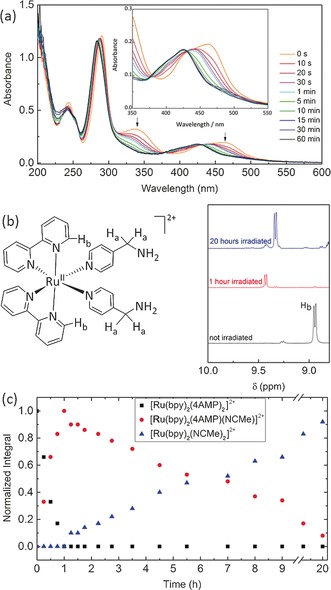
a) Change of the absorption spectrum of [Ru(bpy)_2_(4AMP)_2_](PF_6_)_2_ in acetonitrile solution upon irradiation with an HBO lamp (cut‐off filter at 395 nm, conc. 10^−7^ mol L^−1^). Inset expansion between 350 and 550 nm. b) Formula of [Ru(bpy)_2_(4AMP)_2_]^2+^ and the change in the NMR shifts of the bipyridine protons H_b_ during irradiation. c) Time‐dependent substitution of 4AMP ligands monitored by ^1^H NMR spectroscopy (quantified by integration of the signals of the H_b_ protons).

**Scheme 1:**
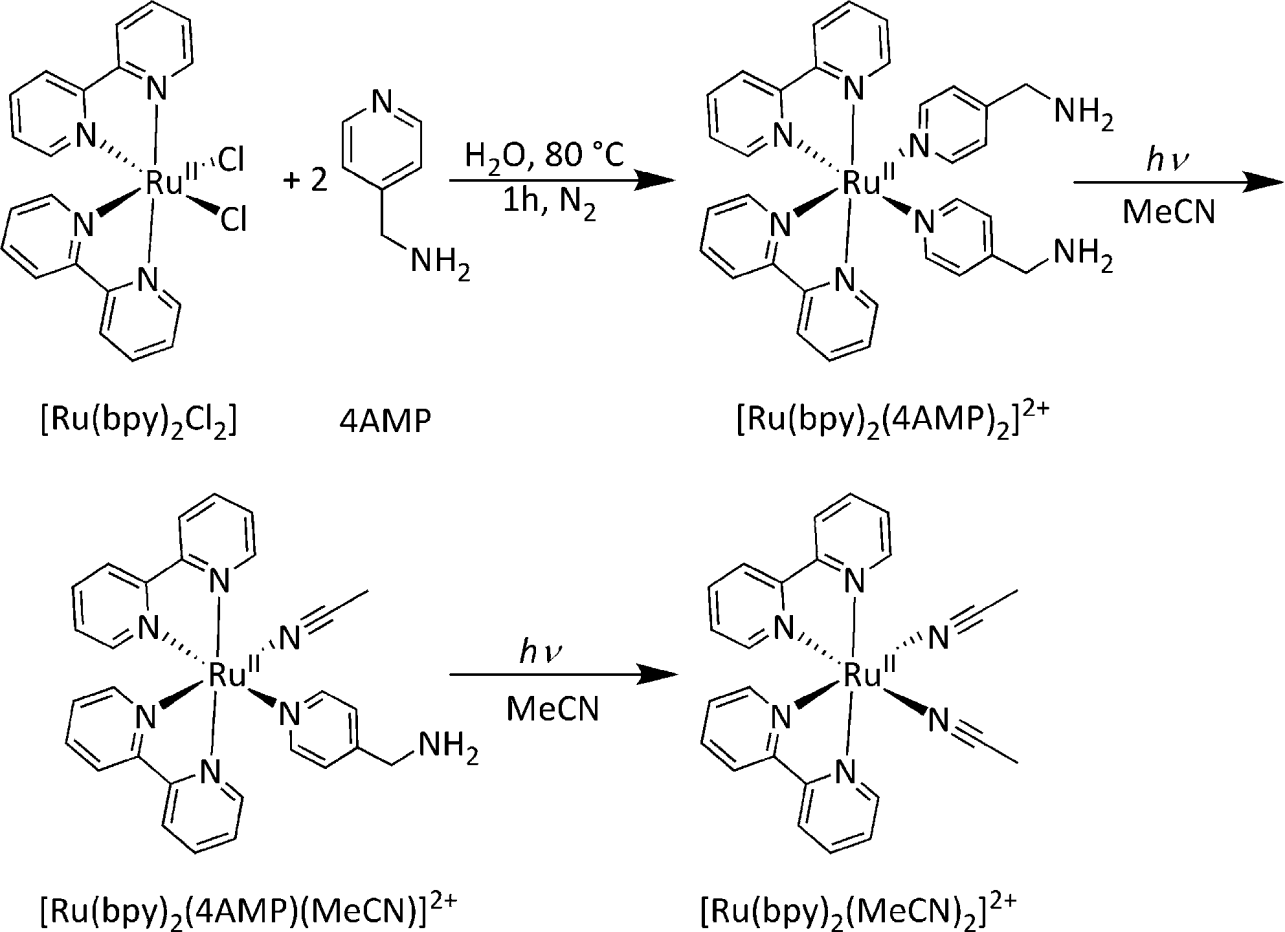
Synthesis and photochemical cleavage of [Ru(bpy)_2_(4AMP)_2_]^2+^. Photolysis was carried out in acetonitrile.

To prepare polymer gels, [Ru(bpy)_2_(4AMP)_2_](PF_6_)_2_ was allowed to react with hexamethylene diisocyanate (HDI) in a 1:1.86 ratio to give a ruthenium‐containing isocyanate‐capped prepolymer (Scheme 2). Chain extension was then achieved with Jeffamine ED‐2003 and as cross‐linker poly(hexamethylene diisocyanate) with about 40 % of the isocyanate groups being contributed by the cross‐linker. Gelation was observed after stirring for around 2 min (Figure 2 a, Figure S10).^21^ The gel was stable in the dark (indeed visibly remained a gel when stored for over 3 months), however, upon irradiation with visible light longer than 395 nm for 10 min the reaction mixture became liquid (Figure 2 b) because of photocleavage at the ruthenium centres. After removing acetonitrile by evaporation, the gel could be swollen in water to give a hydrogel, which subsequently also underwent de‐gelation upon irradiation. Interestingly, photocleavage only occurred in directly irradiated regions, thus opening the possibility for spatial and temporal control to the photocleavage reaction (Figure S11).

**Figure 2:**
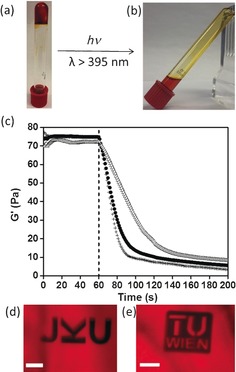
a) Photograph of the gel in MeCN. The gel remains stable when not exposed to light. (A video capturing this process is in the Supporting Information.) b) Photograph taken after irradiation for 10 min at *λ*>395 nm. Cleavage of the pyridyl ligands results in de‐gelation of the gel. c) Light‐induced de‐gelation of the hydrogel measured by real‐time photorheology (light starts after 60 s: ‐ ‐ ‐ ‐; ▵ 10 mW cm^−2^; • 25 mW cm^−2^; +35 mW cm^−2^, all at 400–500 nm). d),e) The hydrogel was micropatterned by means of two‐photon excitation at 800 nm. Scale bar=100 μm. 3D visualizations of these confocal *z*‐stack are available in the Supporting Information.

**Scheme 2:**
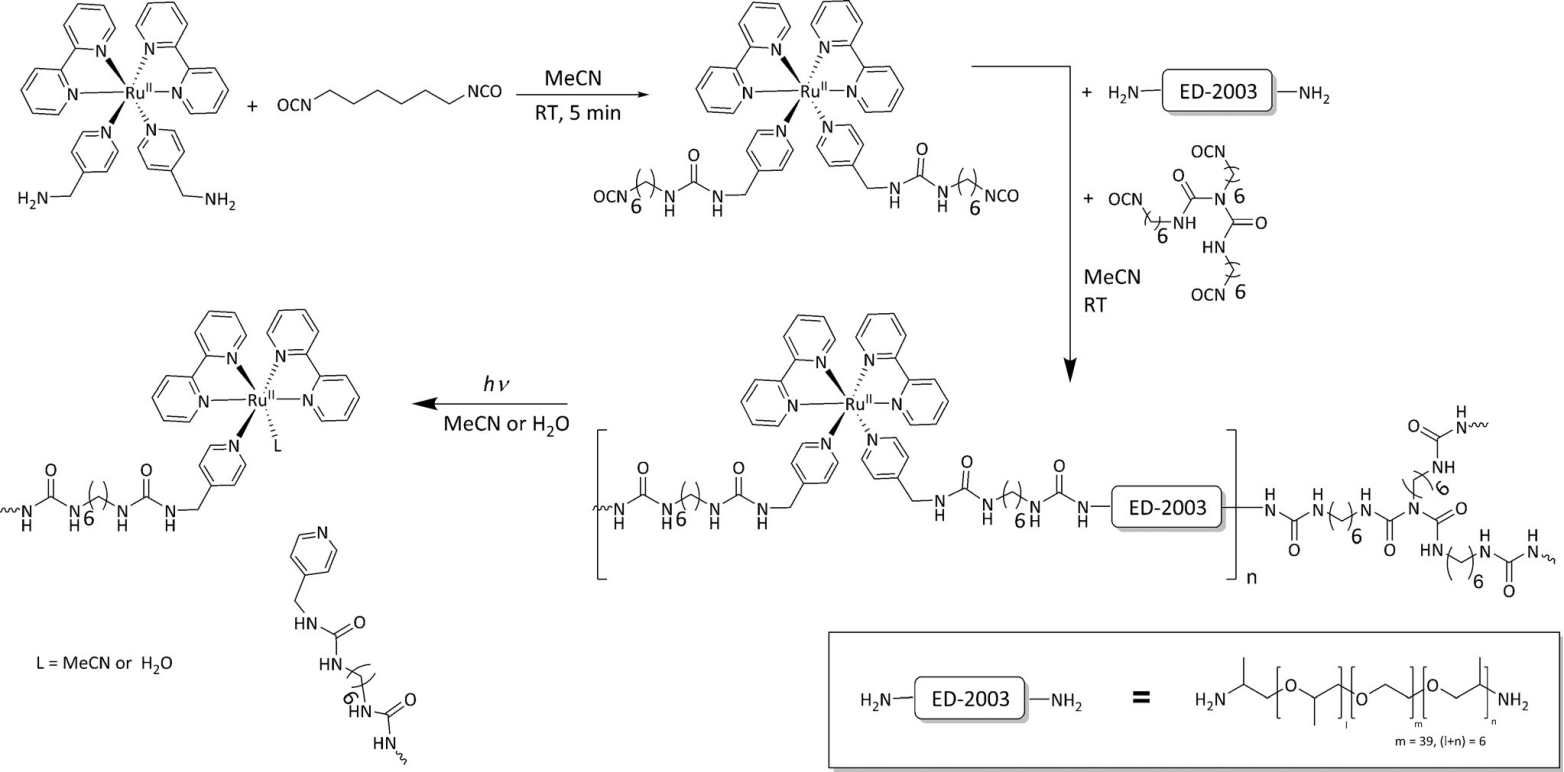
Synthesis and photochemical cleavage of the ruthenium‐containing gel.

To further investigate the de‐gelation process, real‐time(RT)‐photorheology experiments were carried out under visible‐light irradiation.^22^ The storage modulus *G*′ of the gels was measured in oscillation mode (1 %, 2 Hz) and then the samples were irradiated with visible light (400–500 nm, 10 mW cm^−2^). De‐gelation of the organogel in MeCN was immediately detected upon irradiation by a fast decrease in modulus, reaching a final level after 10 s irradiation. The respective hydrogel also showed fast de‐gelation upon irradiation albeit slower than the MeCN‐based organogel (ca. 2 min; Figure S12). The de‐gelation is directly correlated to the photocleavage initiated by the projected visible light, which is further confirmed by irradiation of the hydrogel at various light intensities (Figure 2 c). With increased light intensities the de‐gelation can be significantly accelerated and is achieved already at about 1 min with a light intensity of 35 mW cm^−2^. Hydrogels with higher water content show slightly faster de‐gelation (Figure S13). All the measured gels showed de‐gelation, however maintained a loss factor under 1 (*G*′′/*G*′) as a result of residual crosslinks from poly(hexamethylene diisocyanate) (Figure S14).

To demonstrate the possibility of NIR‐triggered photodegradation, the ruthenium‐containing gels were micropatterned by focused NIR‐laser light in a two‐photon erosion process using a home‐built two‐photon microfabrication setup.^23^ Three‐dimensional micro‐scale logos were carved into the aqueous hydrogels. Visualization of the photo‐eroded areas can be performed by light microscopy using a filter (cut off <520 nm; Figure S15) or by confocal laser scanning microscopy using a 555 nm laser for excitation of auto‐luminescence (Figures 2 d,e). However, this method is invasive causing degradation of the irradiated areas.

In conclusion, a novel complex, [Ru(bpy)_2_(4AMP)_2_](PF_6_)_2_, could be prepared and shown to undergo rapid photocleavage in MeCN or water under visible light. Relatively simple polyurea supramolecular gels were prepared and shown to be stable in the dark or in the absence of solvent, but undergo rapid photocleavage in a spatiotemporally controlled manner upon irradiation as a result of cleavage of the ruthenium–pyridine bonds in the polymer main chain. Photocleavage occurred at wavelengths longer than 395 nm as well as via a two‐photon process in the NIR region (800 nm). Thus, the potential of such ruthenium‐based photocleavable complexes as building blocks for advanced photoresponsive polymeric materials has been demonstrated.

## Conflict of interest

The authors declare no conflict of interest.

## Supporting information

As a service to our authors and readers, this journal provides supporting information supplied by the authors. Such materials are peer reviewed and may be re‐organized for online delivery, but are not copy‐edited or typeset. Technical support issues arising from supporting information (other than missing files) should be addressed to the authors.

Supplementary

Supplementary

Supplementary

Supplementary
