# Supplementary material for: Metallo‐Supramolecular Gels that are Photocleavable with Visible and Near‐Infrared Irradiation
Source: Angew Chem Int Ed Engl. 2017 Nov 15;56(50):15857–60. doi: 10.1002/anie.201707321 (PMC5725706; doi:10.1002/anie.201707321)
Supplement: Supplementary file 3 — Supplementary [file ANIE-56-15857-s003.pdf]

Angewandte Chemie
Eine Zeitschrift der Gesellschaft Deutscher Chemiker

Supporting Information

Metallo-Supramolecular Gels that are Photocleavable with Visible and Near-Infrared Irradiation

Sabrina Theis, Aitziber Iturmendi, Christian Gorsche, Marco Orthofer, Markus Lunzer, Stefan Baudis, Aleksandr Ovsianikov, Robert Liska, Uwe Monkowius, and Ian Teasdale**

anie_201707321_sm_miscellaneous_information.pdf
anie_201707321_sm_Degelation_in_Vis_light.mp4
anie_201707321_sm_JKU_Logo_Render.mov
anie_201707321_sm_TU_Logo_Render.mov

Table of Contents

Table of Contents 1
1. General Experimental Section 2
2. General Experimental Procedure 2
2.1 Synthesis of $[Ru(bpy)_2(4AMP)_2](PF_6)_2$ 2
Synthesis of Ru-Urea Gel 2
2.3 Real time(RT)-photorheology 3
2.4 Two-photon Micropatterning 3
3. Characterization of $[Ru(bpy)_2(4AMP)_2]^{2+}$ 4
3.1 ^{1}H NMR Spectrum 4
3.2 $^{13}C\{^{1}H\}$ NMR Spectrum 5
3.3 HR MS Spectrum 6
3.4 Stability Test 8
3.5 Emission Spectrum 10
3.6 Irradiation experiments of the complex 11
3.7 Irradiation experiments in water 12
4. Ru-Urea Gel 14
4.1. IR-spectrum of the Ru-Urea Gel 14
4.2 Irradiation of the Ru-Urea Gel 15
4.2 Real time (RT)-photorheology of the Ru-Urea Gel and the Hydrogel 16
4.3 Two-photon Micropatterning of the Hydrogel 18
References 18
Author Contributions 19

1. General Experimental Section

All reagents were commercially obtained and used as received. Solvents were purchased from Merck and VWR. Acetonitrile was distilled prior to use. $Ru(bpy)_2Cl_2$ was synthesized according to the literature procedure.[1] All other chemicals were purchased from Sigma Aldrich, Acros Organics and ABCR. The syntheses of the Ru-Urea gel were performed under an atmosphere of nitrogen and in the dark. NMR spectra were obtained with a Bruker Avance III 300 MHz spectrometer. UV-vis spectra were recorded on a Cary 300 Bio photometer. The samples were irradiated with a HBO lamp with 100 W and a cut off filter of 395 nm (GG 395 nm from SCHOTT). ATR-FTIR spectra were recorded on a Perkin Elmer Spectrum 100 FTIR spectrometer equipped with an ATR accessory.

2. General Experimental Procedure

2.1 Synthesis of $[Ru(bpy)_2(4AMP)_2](PF_6)_2$

$[Ru(bpy)_2(4AMP)_2](PF_6)_2$. $Ru(bpy)_2Cl_2$ (300 mg, 0.620 mmol) was suspended in 15 mL of water. The suspension was stirred at 80 °C under N_2 and after dissolution, 4-(aminomethyl)pyridine (4AMP, 350 µL, 3.45 mmol) was added. The reaction mixture was heated for 60 min. After cooling to room temperature the compound was precipitated with a saturated aqueous solution of KPF_6. The orange powder was washed with water and dried. (Yield: 75.8%).
MS (ESI): m/z = 315.1 $[Ru(bpy)_2(4AMP)_2]^{2+}$ and 775.1 $[Ru(bpy)_2(4AMP)_2](PF_6)^+$
^{1}H-NMR (300.15 MHz, acetonitrile-d_3): δ 8.91 (d, J = 5.55 Hz, 2 H_d), 8.35 (d, J = 7.95 Hz, 2 H_g), 8.27 (d, J = 7.95 Hz, 2 H_h), 8.14 (m, 2 H_j and 4 H_b), 7.90 (m, 2 H_e and 2 H_f), 7.76 (t, J = 6.61 Hz, 2 H_i), 7.35 (t, J = 6.88 Hz, 2 H_k), 7.26 (d, J = 5.74 Hz, 4 H_c), 3.80 (s, 4 H_a), 1.49 (s, 4 $–NH_2$).
$^{13}C\{^1H\}$-NMR (75.47 MHz, acetonitrile-d_3): δ 158.18, 158.13, 156.71, 153.39, 153.27, 152.90, 138.33, 138.00, 128.37, 124.80, 128.08, 124.46, 124.19, 44.44.

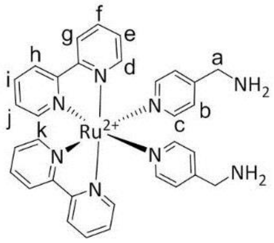

Synthesis of Ru-Urea Gel

Ru-Urea Gel. $[Ru(bpy)_2(4AMP)_2](PF_6)_2$ (23 mg, 25 µmol) was dissolved in 0.3 mL of acetonitrile. Hexamethylene diisocyanate (HDI, 7 µL, 44 µmol) was added and the reaction mixture was stirred for five minutes. Jeffamine® ED-2003 (90 mg, 47 µmol) and poly(hexamethylene diisocyanate) (9.3 mg, 19 µmol) were dissolved in 0.3 mL of acetonitrile. The solutions were combined and stirred until an orange gel is obtained. The amounts were chosen to maintain an overall equimolar stoichiometric ratio of the amine (0.35 eq. ruthenium complex and 0.65 eq. ED-2003) and the isocyanate groups (0.4 eq. poly(hexamethylene diisocyanate and 0.6 eq. HDI). Subsequent hydrogels were formed by lyophilization and addition of 0.6 mL H_2O.

2.3 Real time(RT)-photorheology

RT-photorheology measurements were conducted on an Anton Paar MCR 302 WESP rheometer equipped with a P-PTD 200/GL Peltier glass plate and a PP8 measuring system. For all measurements the previously formed gel was applied on the center of the glass plate and then a measurement gap of 200 µm (Ø = 8 mm) was set. Excess gel was removed from the side of the measuring system and the temperature was set to 20 °C. Rheological measurements were conducted in oscillation mode with a strain of 1% and a frequency of 2 Hz. After an initial measurement period of 60 s the materials were irradiated from the underside of the glass plate using an Exfo Omnicure S 2000 with a broadband Hg-lamp and a double waveguide to ensure homogeneous irradiation (300 s, 400-500 nm, exact irradiation intensities were set on the surface of the sample). The irradiation intensity was assessed using an Ocean Optics USB 2000+ spectrometer. During irradiation the storage G′ and loss moduli G″ were recorded with a frequency of 2 Hz for the first 2 minutes and then 1 Hz for the remaining period of irradiation. The derived values for G′ and G″ are not absolute as adhesion of the sample to the measurement plates has an influence and the experimental set-up only allows for rather small sample thickness, due to the limit of light penetration and resulting gradient in rheological information for samples thicker than 500 µm. All degelation experiments were performed in triplicate and showed good reproducibility.

2.4 Two-photon Micropatterning

Two-photon micropatterning of ruthenium containing gels was performed on a home-build two-photon microfabrication setup. Details of the experimental setup were reported previously.[2] Briefly, the setup is equipped with a femtosecond laser oscillator (MaiTai DeepSee, Spectra Physics) operating at 800 nm, which was focused through a water-immersion microscopy objective (32x/0.85 W, Zeiss) delivering a pulse length of 70 fs after the objective.
Preformed gel samples were placed on glass-bottom µ-dishes (35 mm, Ibidi GmbH, Germany). To saturate the atmosphere within the µ-dishes and prevent evaporation of solvent from the gel samples a piece of tissue soaked in the corresponding solvent (acetonitrile or water) was added to the dish. Three-dimensional TU Wien (200 mm x 200 mm x 50 µm) and JKU (300 µm x 100 µm x 50 µm) logos were photocleaved into the hydrogel operating the two-photon microfabrication system at the following parameters: laser power after objective: 50-100 mW, scanning speed: 150 mm s^{-1}, line spacing: 0.1 µm, layer spacing: 0.5 µm. The remaining gel around the photocleaved areas was visualized immediately after structuring by means of light microscopy using a filter (cut off < 520 nm) and laser scanning microscopy (Zeiss LSM 700 and ZEN11 software for evaluation) using a 555 nm laser for excitation of auto-luminescence, which leads to bleaching/degradation of the gel.

3. Characterization of $[Ru(bpy)_2(4AMP)_2]^{2+}$

3.1 ^{1}H NMR Spectrum

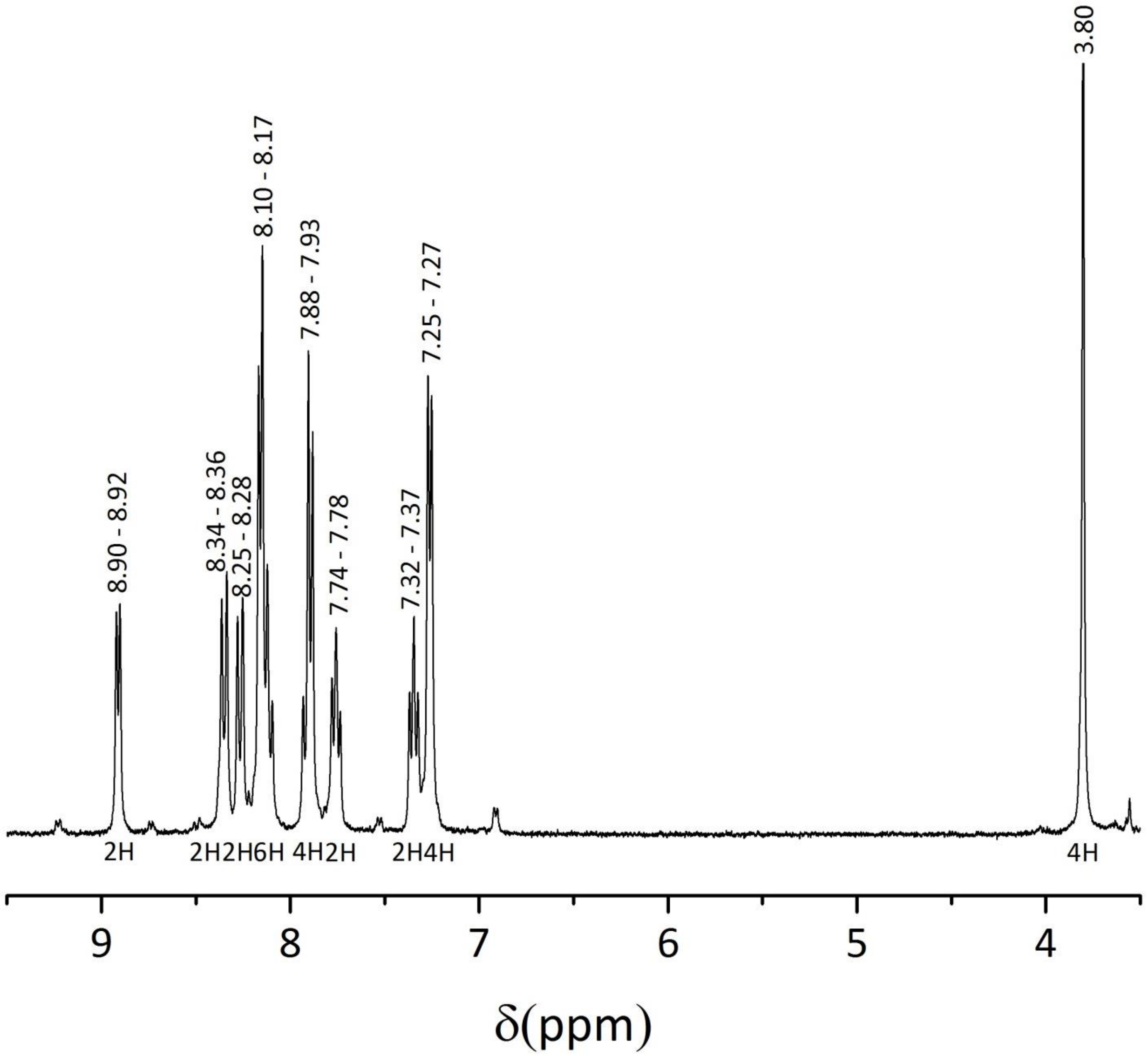

Figure S1. ^{1}H NMR spectrum of $[Ru(bpy)_2(4AMP)_2](PF_6)_2$ in acetonitrile-d_3.

3.2 $^{13}C\{^1H\}$ NMR Spectrum

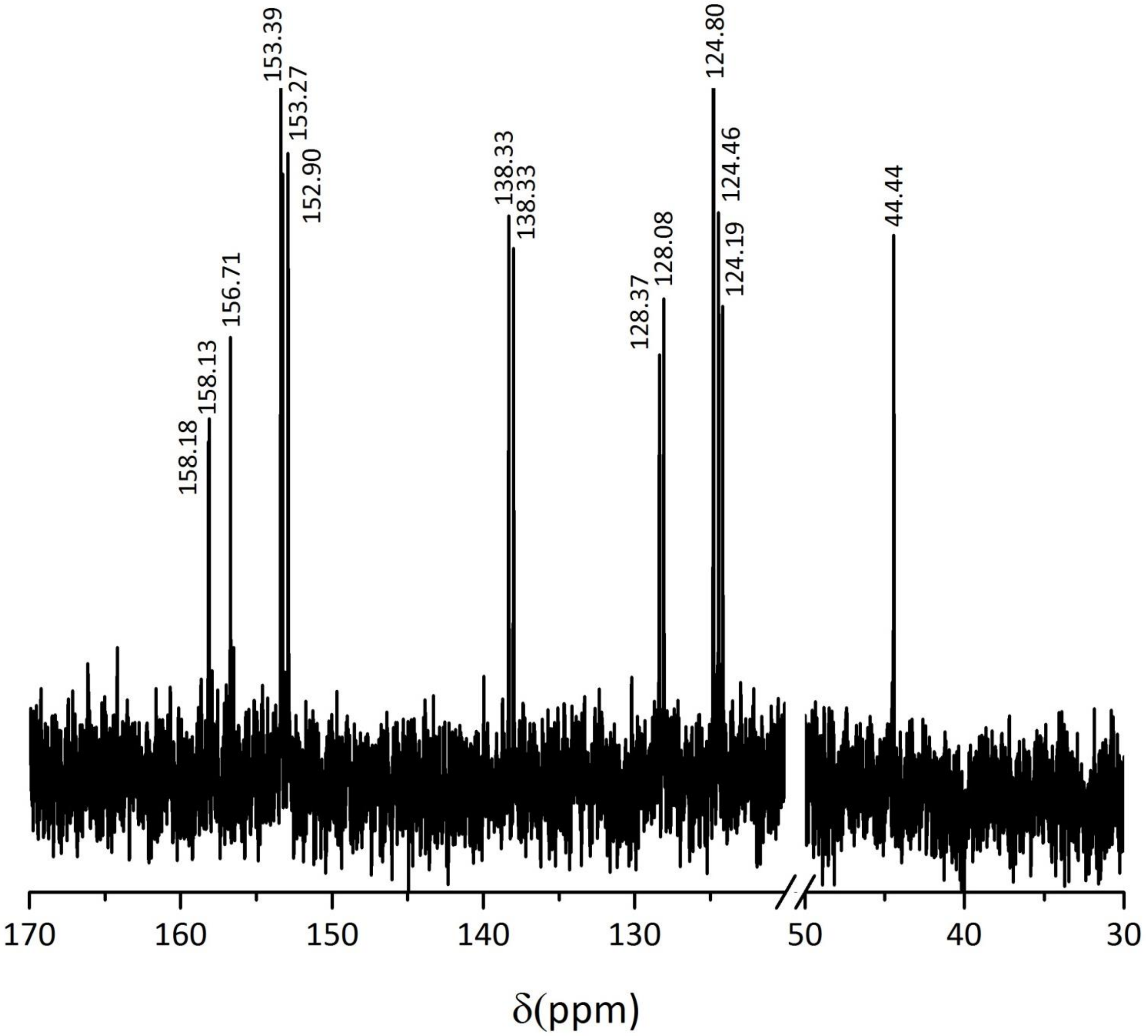

Figure S2. $^{13}C\{^1H\}$ NMR spectrum of $[Ru(bpy)_2(4AMP)_2](PF_6)_2$ in acetonitrile-d_3.

3.3 HR MS Spectrum

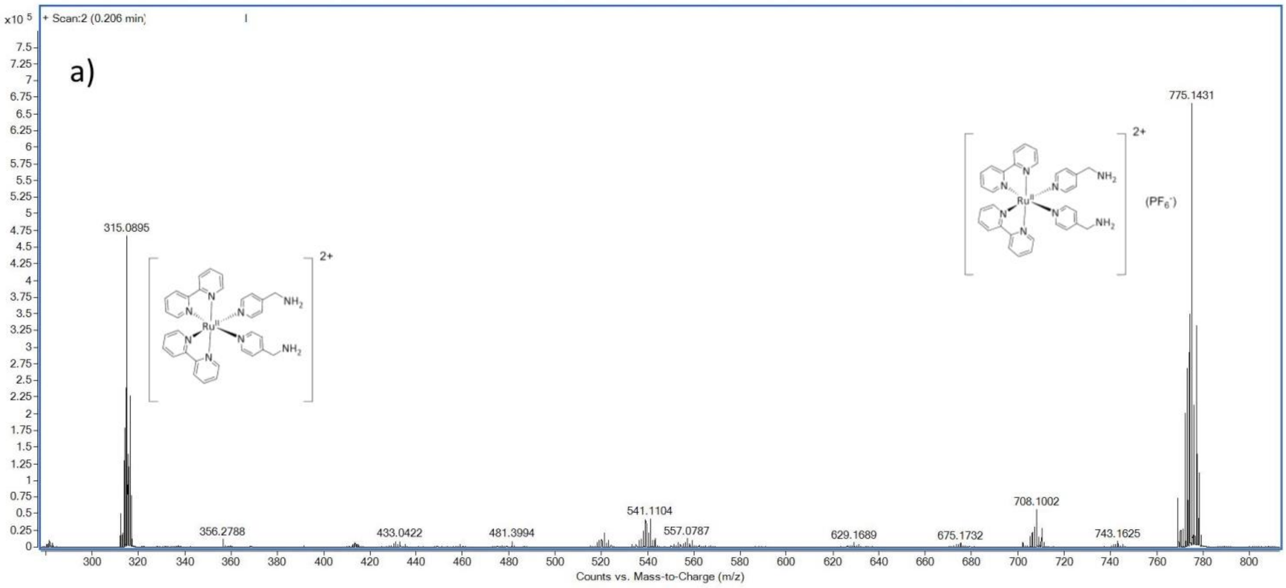

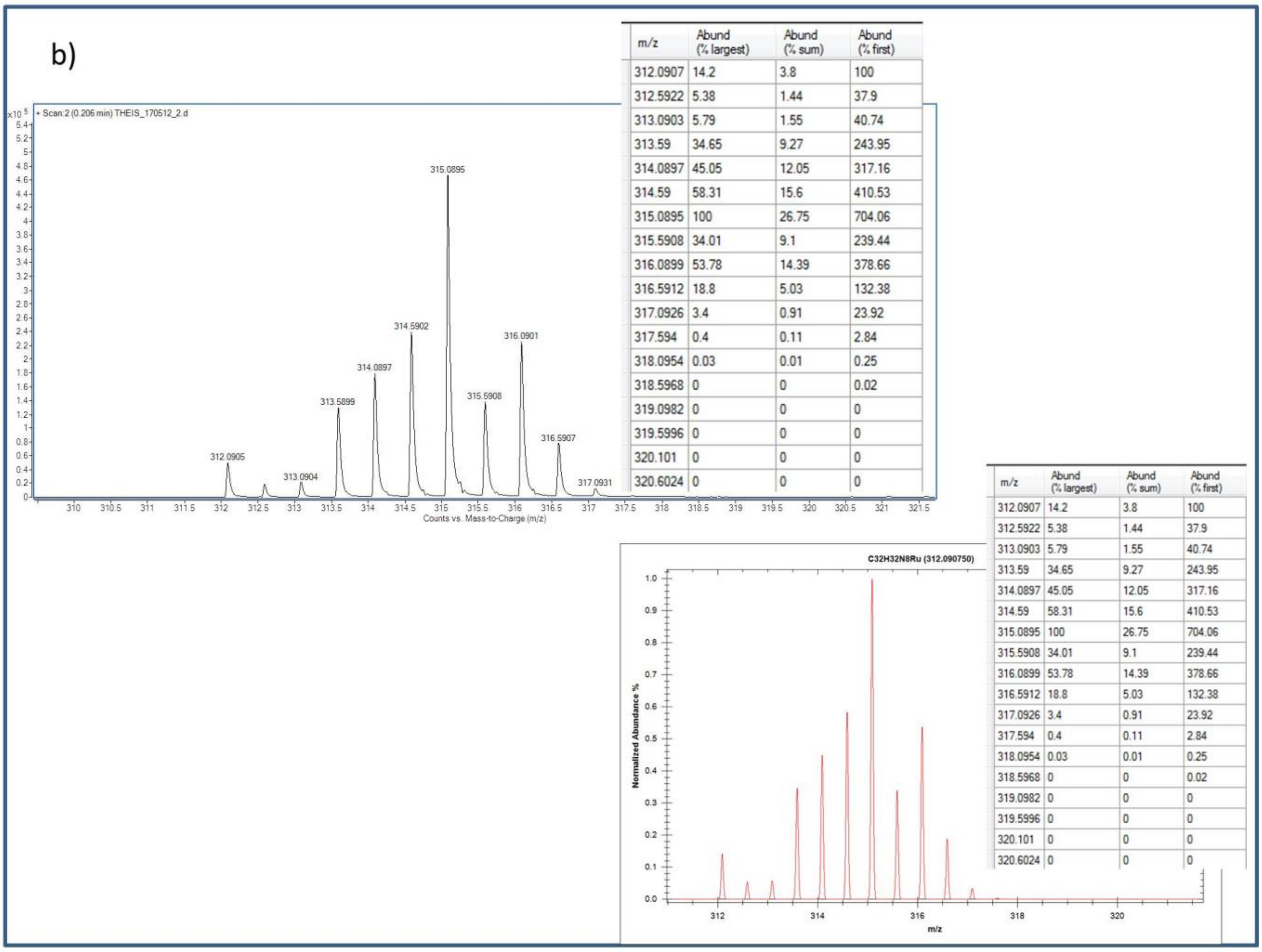

m/z	Abund (% largest)	Abund (% sum)	Abund (% first)
312.0907	14.2	3.8	100
312.5922	5.38	1.44	37.9
313.0903	5.79	1.55	40.74
313.59	34.65	9.27	243.95
314.0897	45.05	12.05	317.16
314.59	58.31	15.6	410.53
315.0895	100	26.75	704.06
315.5908	34.01	9.1	239.44
316.0899	53.78	14.39	378.66
316.5912	18.8	5.03	132.38
317.0926	3.4	0.91	23.92
317.594	0.4	0.11	2.84
318.0954	0.03	0.01	0.25
318.5968	0	0	0.02
319.0982	0	0	0
319.5996	0	0	0
320.101	0	0	0
320.6024	0	0	0

m/z	Abund (% largest)	Abund (% sum)	Abund (% first)
312.0907	14.2	3.8	100
312.5922	5.38	1.44	37.9
313.0903	5.79	1.55	40.74
313.59	34.65	9.27	243.95
314.0897	45.05	12.05	317.16
314.59	58.31	15.6	410.53
315.0895	100	26.75	704.06
315.5908	34.01	9.1	239.44
316.0899	53.78	14.39	378.66
316.5912	18.8	5.03	132.38
317.0926	3.4	0.91	23.92
317.594	0.4	0.11	2.84
318.0954	0.03	0.01	0.25
318.5968	0	0	0.02
319.0982	0	0	0
319.5996	0	0	0
320.101	0	0	0
320.6024	0	0	0

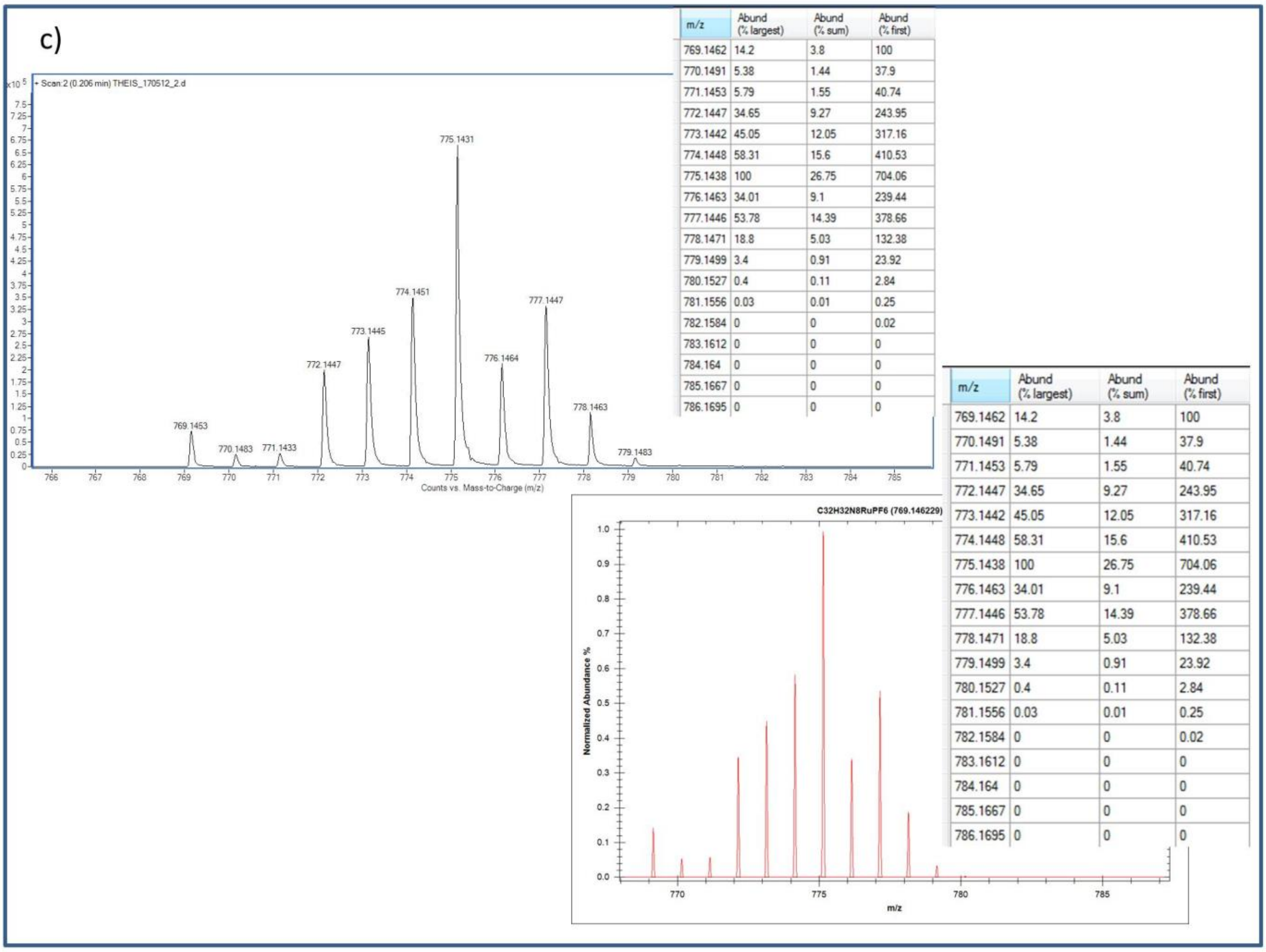

Figure S3. a) HR MS spectrum of $[Ru(bpy)_2(4AMP)_2](PF_6)_2$ in acetonitrile (m/z = 315.1 represents $[Ru(bpy)_2(4AMP)_2]^{2+}$ and m/z = 775.1 represents $\{[Ru(bpy)_2(4AMP)_2](PF_6)\}^{+}$). b) Enlarged peak at m/z = 315.1 and simulated peak. c) Enlarged peak at m/z = 775.1 and simulated peak.

3.4 Stability Test

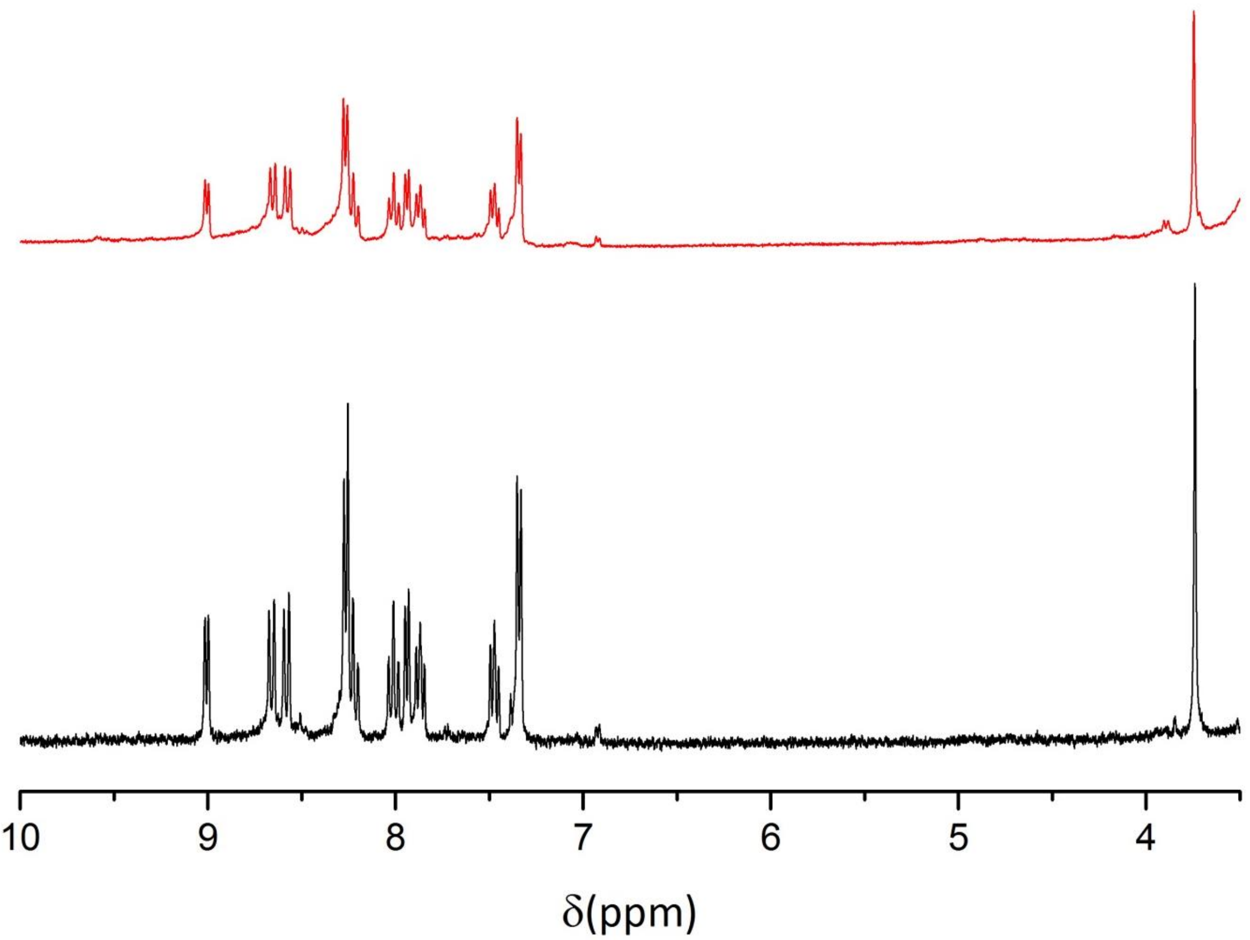

Figure S4. ^1H NMR spectra of complex $[Ru(bpy)_2(4AMP)_2](PF_6)_2$ (in acetonitrile-*d_3*) which was stored in solid state in the dark for >3 months (top) and in solid state exposed to sunlight for several months (bottom).

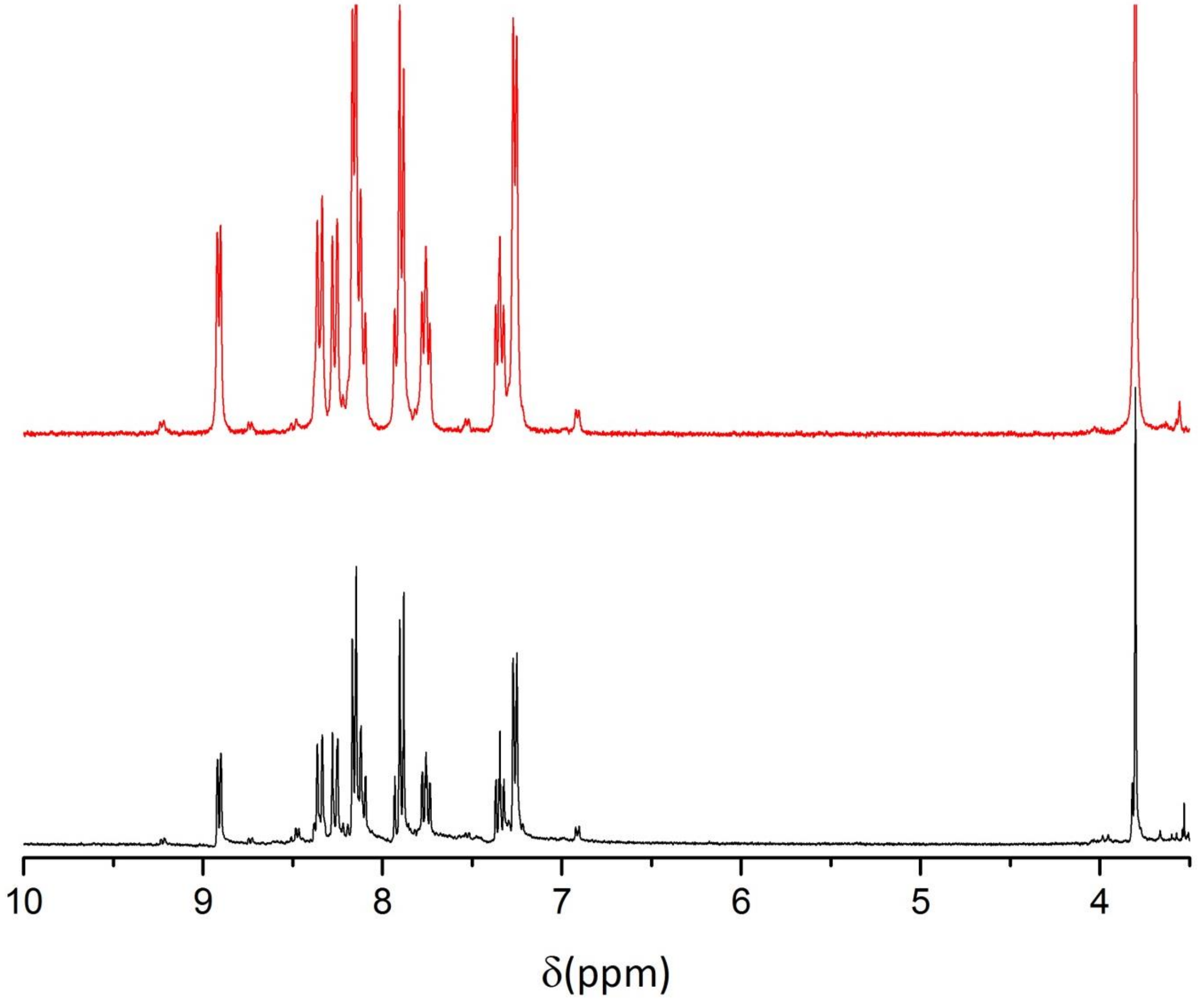

Figure S5. ^{1}H NMR spectra of $[Ru(bpy)_2(4AMP)_2](PF_6)_2$ dissolved in acetonitrile-d_3 before (top) and after (bottom) >3 months in the dark.

3.5 Emission Spectrum

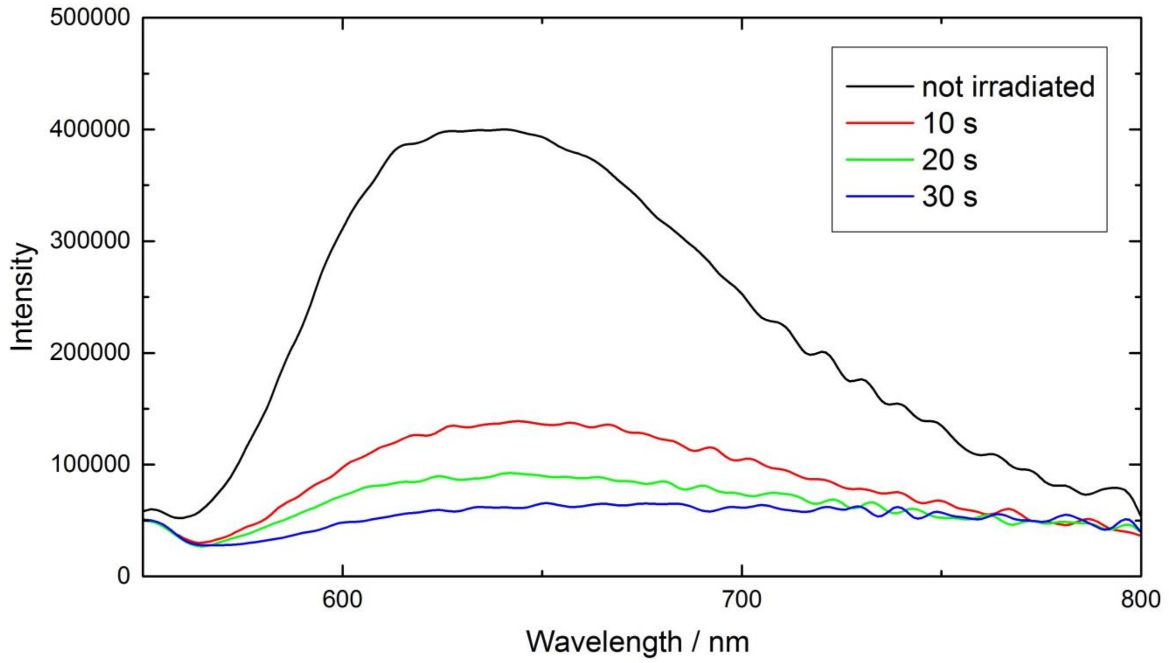

Figure S6. Change of the emission spectra of $[Ru(bpy)_2(4AMP)_2](PF_6)_2$ upon irradiation in de-gassed ethanol. However, due to the low intensity and the general oxygen sensitivity of the phosphorescence, emission spectroscopy is not a good tool to follow this photo-substitution reaction.

3.6 Irradiation experiments of the complex

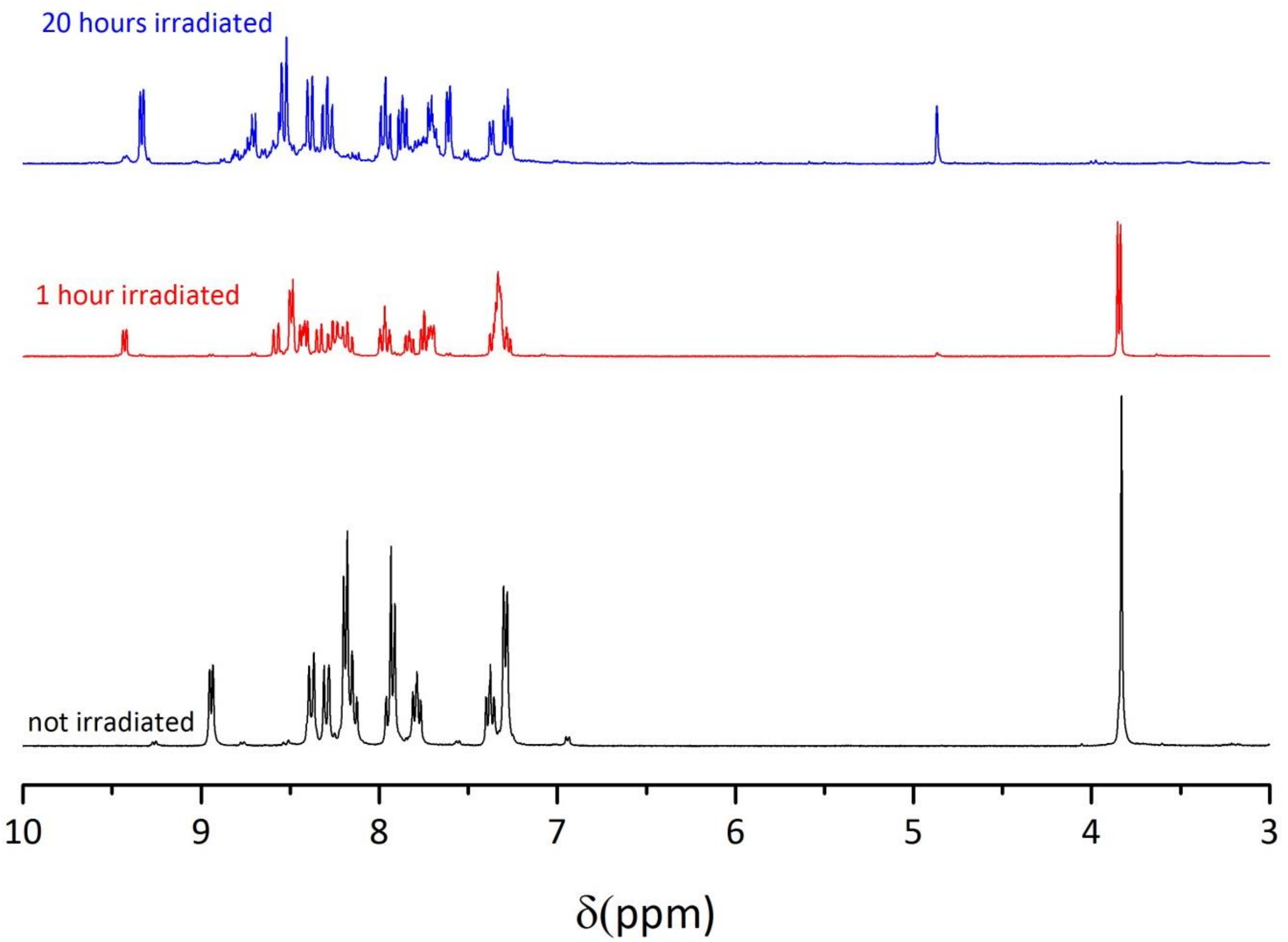

Figure S7. ^{1}H NMR spectra of $[Ru(bpy)_2(4AMP)_2](PF_6)_2$ in acetonitrile-d_3 during irradiation.

3.7 Irradiation experiments in water

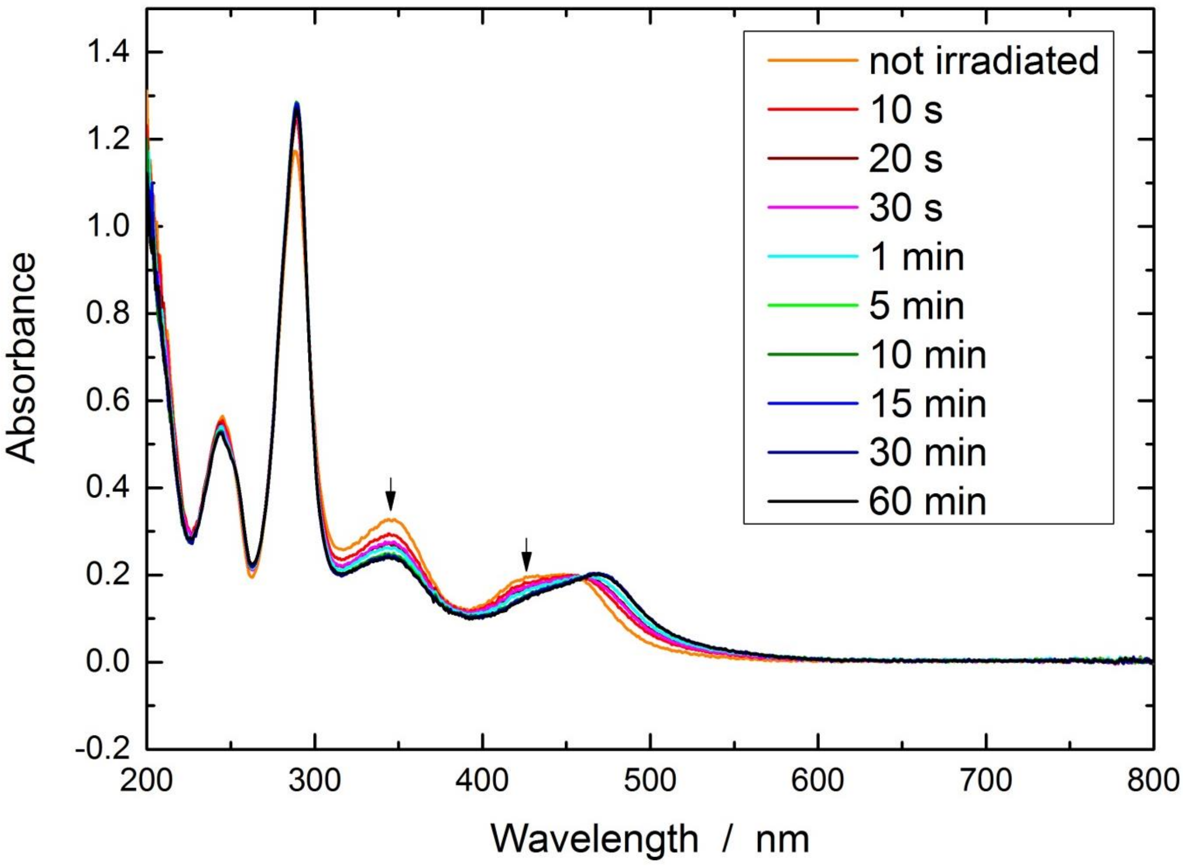

Figure S8. Change of the absorption spectra of $[Ru(bpy)_2(4AMP)_2](PF_6)_2$ upon irradiation with light >395 nm in H_2O. Compared to the photo-substitution in acetonitrile, the reaction in water is slower and the isosbestic point does not vanish even after elongated irradiation. This is an indication for a clean one-step reaction within the irradiation time with only one 4AMP ligand being cleaved. The NMR spectra in D_2O supports this interpretation (see Figure S9).

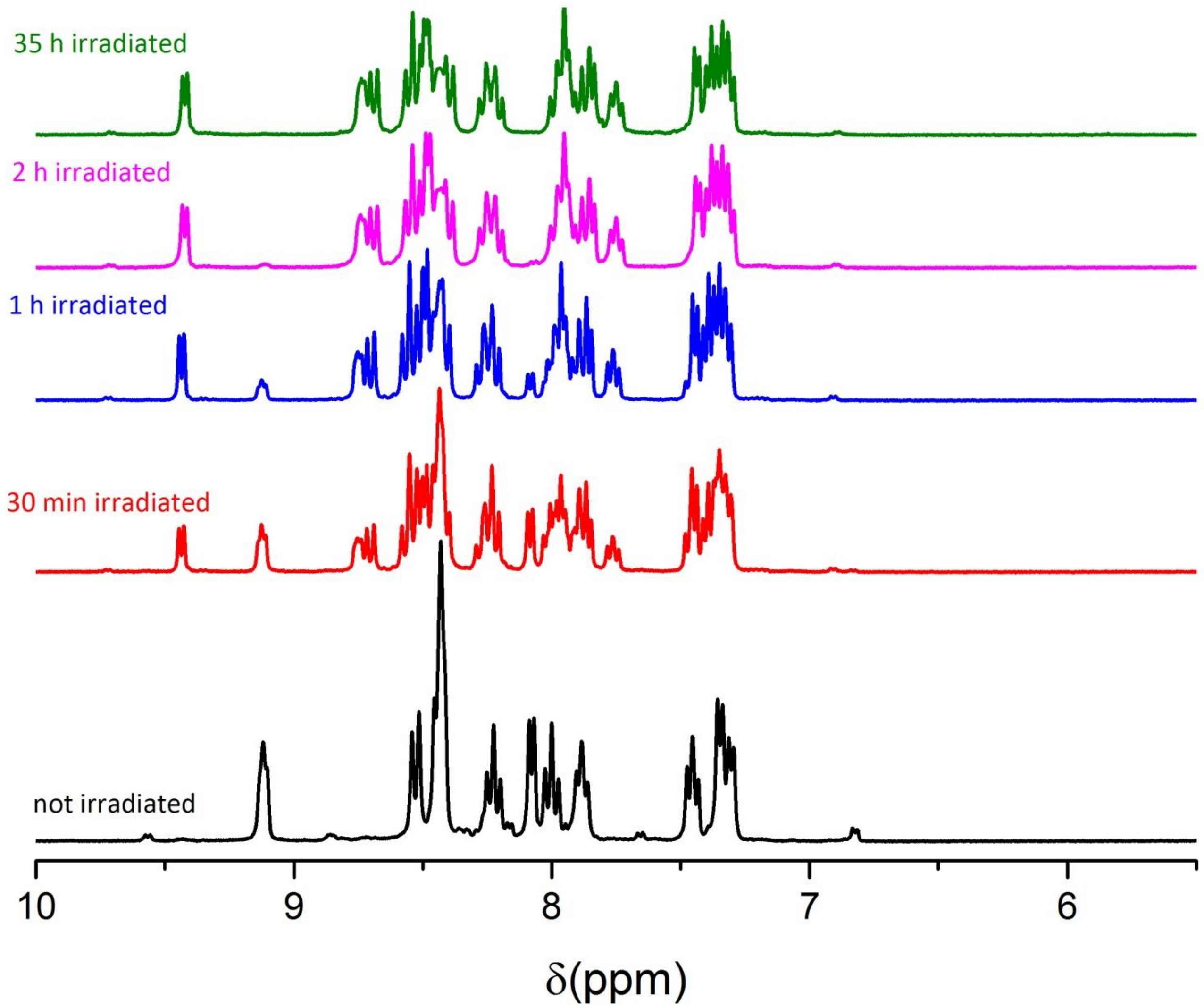

Figure S9. ^{1}H NMR spectra of $[Ru(bpy)_2(4AMP)_2](PF_6)_2$ dissolved in two drops of acetone-d_6 and measured in D_2O during irradiation.

4. Ru-Urea Gel

4.1. FTIR-spectrum of the Ru-Urea Gel

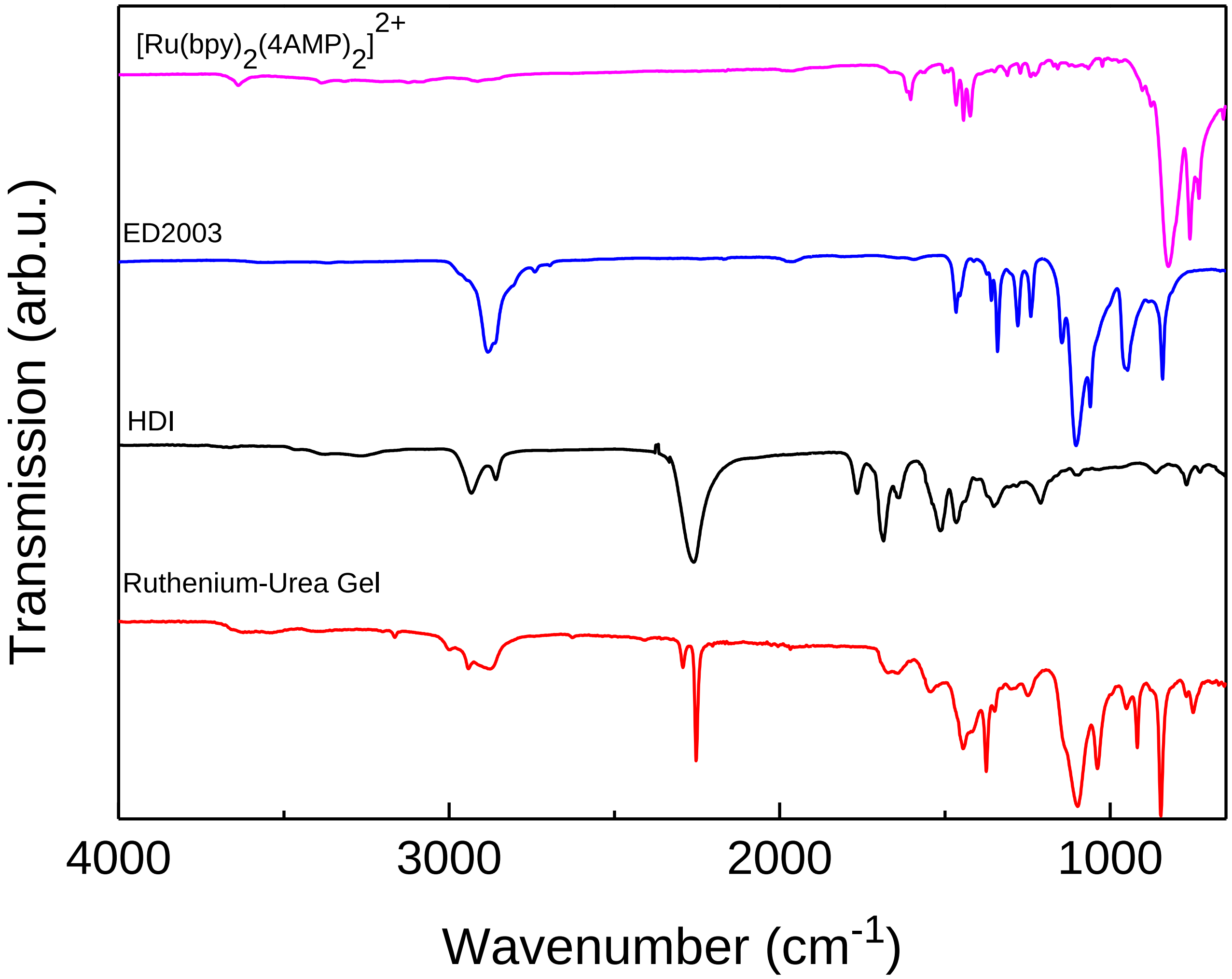

Figure S10. ATR-IR spectra of the starting materials and the resulting gel. The –NH_2 signal of 4AMP moiety of the Ru complex at 1605 cm^{-1} vanishes, thus suggesting high conversion. The intense isocyanate stretching of -NCO at 2260 cm^{-1} also vanishes indicating complete crosslinker conversion. The signals at 2299 and 2254 cm^{-1} and below 1500 cm^{-1} are assigned to free acetonitrile in the gel.[3] Urea C=O has signals between 1600-1800 cm^{-1} depending on its substituents, presence of hydrogen bonding and crystallinity of the material.[4] Hence, the broad bands between 1620-1705 cm^{-1} are assigned to C=O stretching vibrations, signals at 1545 cm^{-1} to δ(N–H).[5]

4.2 Irradiation of the Ru-Urea Gel

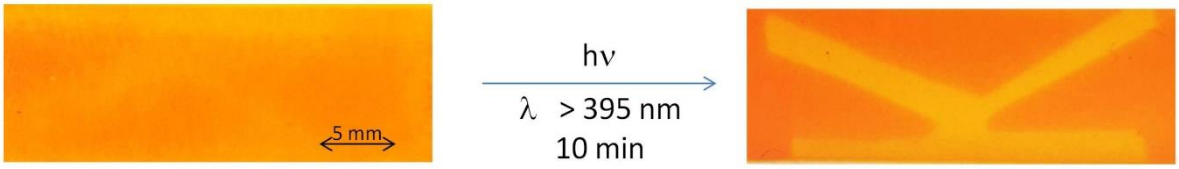

Figure S11. Left: not irradiated gel. Right: Irradiation with visible light (λ > 395 nm) for 10 minutes using of a cardboard mask.

4.2 Real time (RT)-photorheology of the Ru-Urea Gel and the Hydrogel

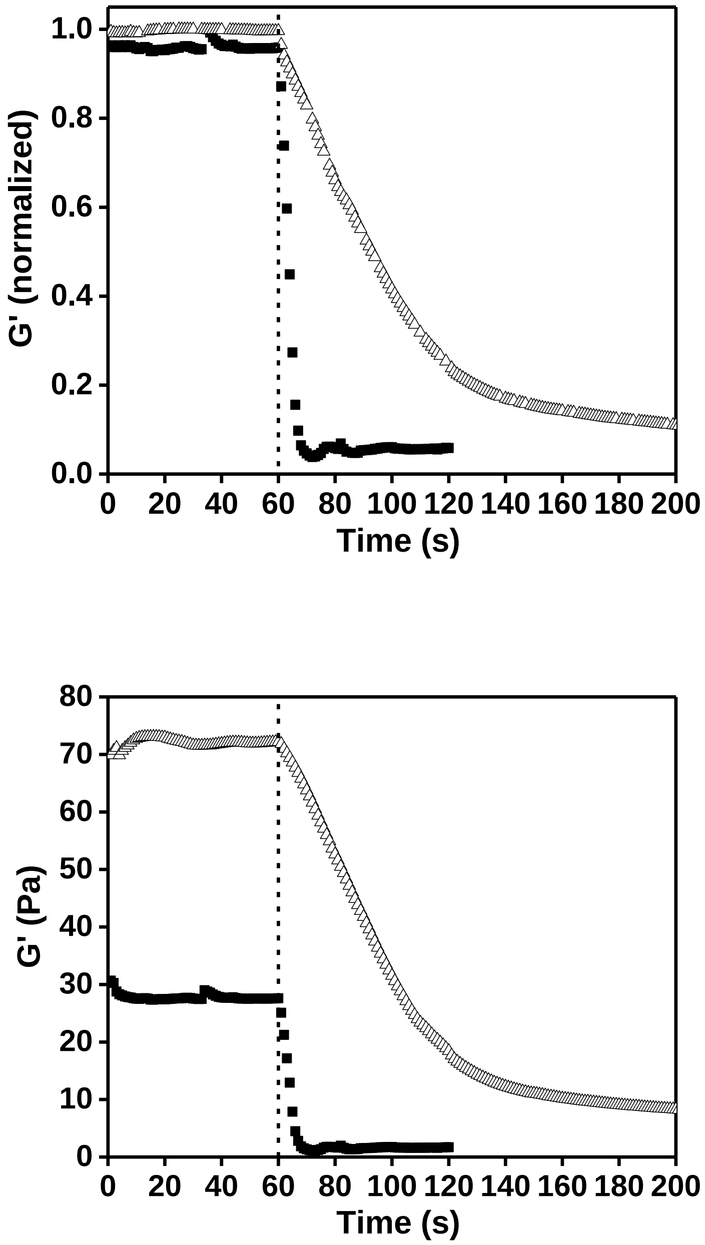

Figure S12. Light-induced de-gelation measured by RT-photorheology (light starts after 60 s: ---; light intensity 10 mW cm^{-2}; ■ organogel in MeCN; ∆ hydrogel). (above normalized data, below actual data).

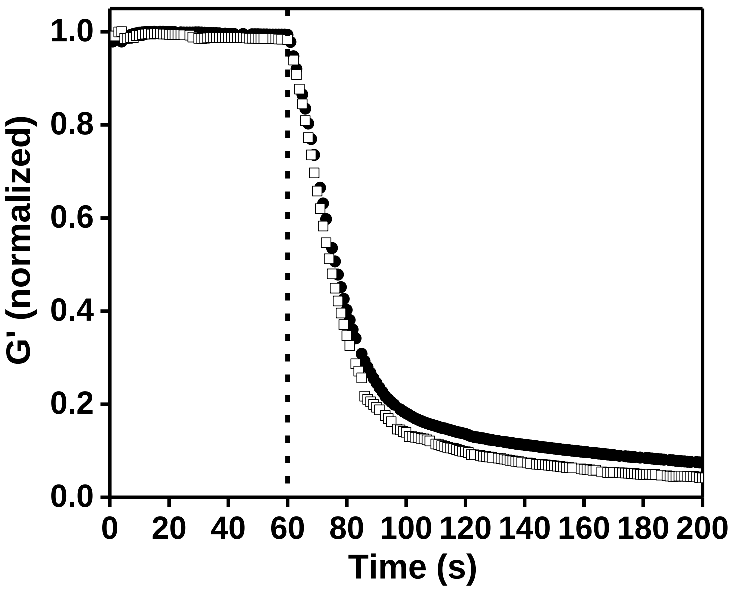

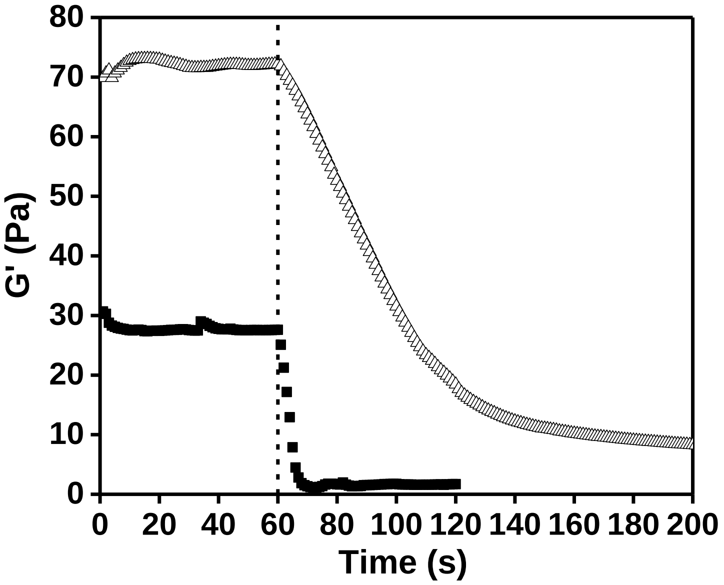

Figure S13. Light-induced de-gelation measured by RT-photorheology (light starts after 60 s: ---; light intensity 25 mW cm^{-2}; ● hydrogel; □ 0.1 mL H_2O added). (above normalized data, below actual data).

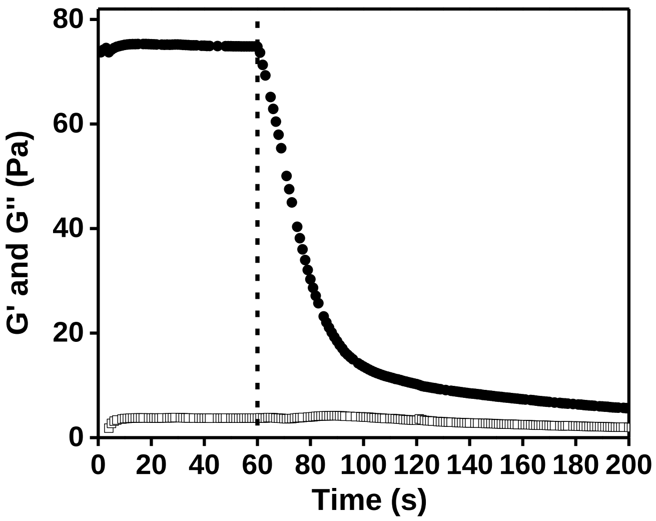

Figure S14. Light-induced de-gelation of the prepared hydrogel measured by RT-photorheology (light starts after 60 s: ---; light intensity 25 mW cm^{-2}; ● storage modulus G′; □ loss modulus G″).

4.3 Two-photon Micropatterning of the Hydrogel

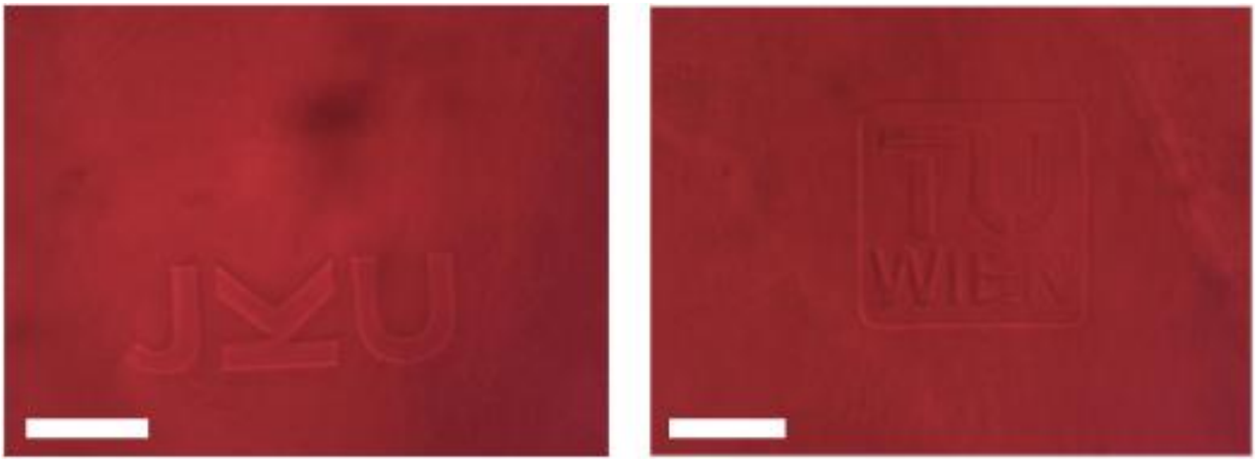

Figure S15: Light microscopy images of the micropatterned logos in the ruthenium containing hydrogel. A filter was used for imaging (cut off < 520 nm). Scale bar = 100 µm.

References

[1] B. P. Sullivan, D. J. Salmon, T. J. Meyer, *Inorg. Chem.* **1978**, *17*, 3334-3341.

[2] A. Ovsianikov, S. Mühleder, J. Torgersen, Z. Li, X.-H. Qin, S. Van Vlierberghe, P. Dubruel, W. Holnthoner, H. Redl, R. Liska, J. Stampfl, *Langmuir* **2014**, *30*, 3787-3794.

[3] Spectral Database for Organic Compounds SDBS, http://sdbs.db.aist.go.jp/sdbs/cgi-bin/direct_frame_top.cgi

[4] L. Ning, W. De-Ning, Y. Sheng-Kang, *Polymer* **37**, 3577-3583.

[5] M. Hesse, H. Meier, B. Zeeh, Spektroskopische Methoden in der organischen Chemie, Thieme, **2005**.

Author Contributions

S. Theis: Experimental synthesis and data collection and manuscript preparation

A. Iturmendi: Supporting polymer synthesis and characterization and manuscript preparation

C Gorsche: Experimental design, data collection of photorheology; co-writing of manuscript

M. Orthofer: Complex preparation

M. Lunzer: Two-photon micropatterning experimental work, microscopy, co-writing of manuscript

S. Baudis: Experimental design two-photon experiments and writing of the manuscript

A. Ovsianikov: Conception of two-photon experiments

R. Liska. Co-conception of gel characterization experiments

U. Monkowius: Conception and experimental design; co-writing of manuscript

I. Teasdale: Funding acquisition and conception; co-writing of manuscript
